# Tele-Rehabilitation to Combat Rehabilitation Service Disruption During COVID-19 in Hong Kong: Observational Study

**DOI:** 10.2196/19946

**Published:** 2021-08-19

**Authors:** Benny Pang Shing Ku, Ada Wai Shan Tse, Benny Chu Hang Pang, Ngai Tseung Cheung, Joanna Yuk Wa Pang, Joyce Ka Yin Chan, Hing Loi Hui, Dave Chu, Kevin Hoi Wa Choi

**Affiliations:** 1 Hospital Authority Information Technology and Health Informatics Hong Kong Hong Kong

**Keywords:** health information technology, mobile app, allied health, tele-rehabilitation, telehealth, rehabilitation, app, COVID-19

## Abstract

**Background:**

A tele-rehabilitation platform was developed to improve access to ambulatory rehabilitation services in Hong Kong. The development was completed in October 2019 and rolled out for use to occupational therapists, physiotherapists, and speech therapists. During the COVID-19 pandemic, rehabilitation services were severely interrupted. Tele-rehabilitation was used extensively to meet the demand for rehabilitation service delivery.

**Objective:**

The aims of this study were to (1) describe the design and development process of a tele-rehabilitation service, and (2) study how the tele-rehabilitation platform was used to overcome the disruption of rehabilitation service during the COVID-19 pandemic.

**Methods:**

Tele-rehabilitation was developed utilizing 4 core determinants of Unified Theory of Acceptance and Use of Technology as guiding principles. A generic prescription platform, called the activity-based prescription system, and a mobile app, called the Rehabilitation App, were built. Five outcomes were used to examine the utilization of tele-rehabilitation both before and during the pandemic: throughput, patient demographic, patient conditions, workforce, and satisfaction from patients and staff.

**Results:**

There was a tremendous increase in the use of tele-rehabilitation during pandemic. The total number of patients (up until July 2020) was 9101, and the main age range was between 51 to 70 years old. Tele-rehabilitation was used for a much wider scope of patient conditions than originally planned. More than 1112 therapists, which constituted 50.6% of the total workforce (1112/2196), prescribed tele-rehabilitation to their patients. Moreover, there was a high satisfaction rate from patients, with a mean rating of 4.2 out of 5, and a high adherence rate to prescribed rehabilitation activities (107840/131995, 81.7%).

**Conclusions:**

The findings of our study suggested that tele-rehabilitation in the form of a generic prescription platform and mobile app can be an effective means to provide rehabilitation to patient. During the COVID-19 pandemic, tele-rehabilitation has been used extensively and effectively to mitigate service disruption. Our findings also provide support that there is a high level of satisfaction with tele-rehabilitation; however, a longer duration study is required to demonstrate the sustained use of tele-rehabilitation, especially after the pandemic.

## Introduction

### Rehabilitation Demand and Service Gap in Hong Kong

The Hospital Authority is the statutory body responsible for managing public health services in Hong Kong. The Hospital Authority provides over 90% of inpatient care and 30% of outpatient care and is the major provider of rehabilitation service to Hong Kong citizens [1]. The Hong Kong Census and Statistics Department projects that the percentage of older adults—individuals over 65 years of age—will increase from 14.7% in 2014 to 23.3% in 2026 [1]. There is heavy demand for rehabilitation services by the aging population. In 2013, there were approximately 18,000 and 6100 acute admissions for stroke and hip fracture, respectively, treated by Hospital Authority [2], and 38% of stroke patients and 70% of hip fracture patients were transferred to extended care hospitals for rehabilitation. The average length of extended care stay for patients after stroke was 34.4 days, while that for patients with hip fracture was 23.9 days [3]. Ambulatory rehabilitation services are provided to patients upon discharge from hospitals. The Hospital Authority commissioned a territory-wide strategic service framework study on rehabilitation service in 2016 and the report indicated that there were serious problems: (1) inadequate ambulatory rehabilitation service placement, (2) long wait times for service, and (3) inadequate therapy intensity and frequency for patients in need [4]. The above-mentioned inadequacies for ambulatory service (1) hindered the flow of patients from acute hospital to extended care hospital, (2) delayed discharge of patients from extended hospital, and (3) became a barrier to patients’ reintegration into the community. In 2018 and 2019, there were over 2.8 million allied health outpatient attendances and over 6 million inpatient and day patient attendances [2]. Stroke, cardiovascular diseases, musculoskeletal diseases or trauma, and respiratory diseases were the 4 major groups requiring intensive rehabilitation services.

To overcome service bottlenecks, especially those for patients after stroke, patients after hip fracture, and older adult patients with frailty, the report [4] recommended the development of tele-rehabilitation and pursued novel service delivery models such as tele-therapy, tele-monitoring, and tele-education. The objective was to improve overall access to rehabilitation services. In line with the recommendations, the Hospital Authority Annual Plan 2019-2020 included the strategic development of mobile solutions to facilitate the public’s access to Hospital Authority service [3,4].

### Hospital Authority Tele-rehabilitation

Tele-rehabilitation refers to the provision of rehabilitation service at a distance using telecommunication technology as the service delivery medium. It is an alternative means of providing all aspects of care including interviews, physical assessments, diagnoses, interventions, maintenance activities, consultations, education, and training to patients in a remote location [5]. Tele-rehabilitation has been practiced overseas for many years, and there is a lot of research indicating its effectiveness for various kinds of conditions [6-11]. There are a number of benefits, both to patient and family, including (1) potential transportation, cost, and time savings; (2) continuity of patient care; (3) the ability for patients to perform interventions at convenient times, intensity, and sequencing; and (4) the positive effect for the patient of performing rehabilitation in their own social and vocational environment [6]. Tele-rehabilitation has been practiced by different allied health professions since its introduction [7-10], but Hong Kong has lagged in the development of tele-rehabilitation—there are relatively few studies because health care providers have not considered the need for tele-rehabilitation in Hong Kong. However, 1 tele-rehabilitation study [11] in Hong Kong demonstrated the feasibility, efficacy, and high level of acceptance of tele-rehabilitation among community-dwelling patients after stroke.

### COVID-19 Outbreak

The tele-rehabilitation platform’s development was completed in October 2019. The aim of its development was to provide therapists with a new form of service delivery. Since mid-January 2020, COVID-19 has affected Hong Kong and in late January 2020 rehabilitation services delivery became seriously disrupted, with a 50% drop in attendance. To combat the disruption of service, occupational therapists, physiotherapists, and speech therapists extensively utilized the tele-rehabilitation platform from mid-February 2020 onward, and its content expanded rapidly from early March 2020 onward, gathering momentum during the COVID-19 outbreak.

The aims of this study were to describe the design and development process of the tele-rehabilitation platform and investigate how the tele-rehabilitation platform was used to overcome the disruption of rehabilitation services during the COVID-19 pandemic.

## Methods

### Theoretical Basis

A technology or innovation can only be considered useful if it is accepted and used in daily clinical practice. There are several criteria to consider in predicting whether target users will actually use the technology. The Unified Theory of Acceptance and Use of Technology [12], based on conceptual and empirical similarities of various technology acceptance models, was used as a framework to guide on the development of the Hospital Authority's tele-rehabilitation platform. The model contained 4 core determinants—(1) performance expectancy (ease of use), (2) effort expectancy (perceived usefulness), (3) organizational facilitating conditions, and (4) social influence—of user’s intention to use and actual use behavior of the new technology [12]. *Performance expectancy* is defined as the degree to which an individual believes that the use of the new system will help them improve their job performance. *Effort expectancy* is defined as the degree to which a person perceives the system as easy to use. *Social influence* is defined as the degree to which an individual perceives that important others believe they should use an information system. *Organizational facilitating condition* is defined as the degree to which an individual believes an organizational and technical infrastructure exists to support the use of the system. These determinants applicable to technology acceptance by health care workers [13]. These 4 guiding principles indicated that the tele-rehabilitation platform should (1) be easy to use; (2) be able to help therapists provide treatment to patients in need; (3) have adequate support and training provided; and (4) be such that the user is well engaged and perceives the importance of its use. By adhering to these principles, we hoped to foster the intention of use and actual use of tele-rehabilitation. The development was user centric and conducted in close collaboration with clinical users and patients.

### Requirements and Design

Focus groups were formed to work in close collaboration with physiotherapists, occupational therapists, and speech therapists. An agile approach was used; therapists could test the prototypes during regular focus group meetings and provide feedback. Moreover, patients were invited to try the mobile app and provide comments on a regular basis for continuous user interface improvements.

Tele-rehabilitation has been used for patients after stroke [14-16], in cardiac rehabilitation [17-20], after total knee replacement and total hip replacement [21-24], with multiple sclerosis [25,26], with aphasia and speech disorders [27-29], with cognitive impairments [30,31], and after hip fracture [32,33]. Tele-rehabilitation modes can be grouped into videoconferencing, virtual reality, sensors (or wearables), and mobile apps [9,10]. Different types of tele-rehabilitation have their own advantages and weaknesses. Randomized controlled trials [21,22,26] have demonstrated clinical evidence supporting the effectiveness of tele-rehabilitation. It was stressed by our users that the tele-rehabilitation platform should (1) bridge the service gap in the ambulatory rehabilitation service, (2) enable the therapists to prescribe suitable exercise to patients, and (3) save time for the therapist in view of their current heavy workload. It was emphasized that patients should be able to carry out prescribed rehabilitation activities anywhere and anytime by themselves. Tele-rehabilitation using off-the-shelf technology was favorable [19,34] because it can be easily accessible to patients (ie, without the need to procure and install sophisticated equipment.)

**Figure 1:**
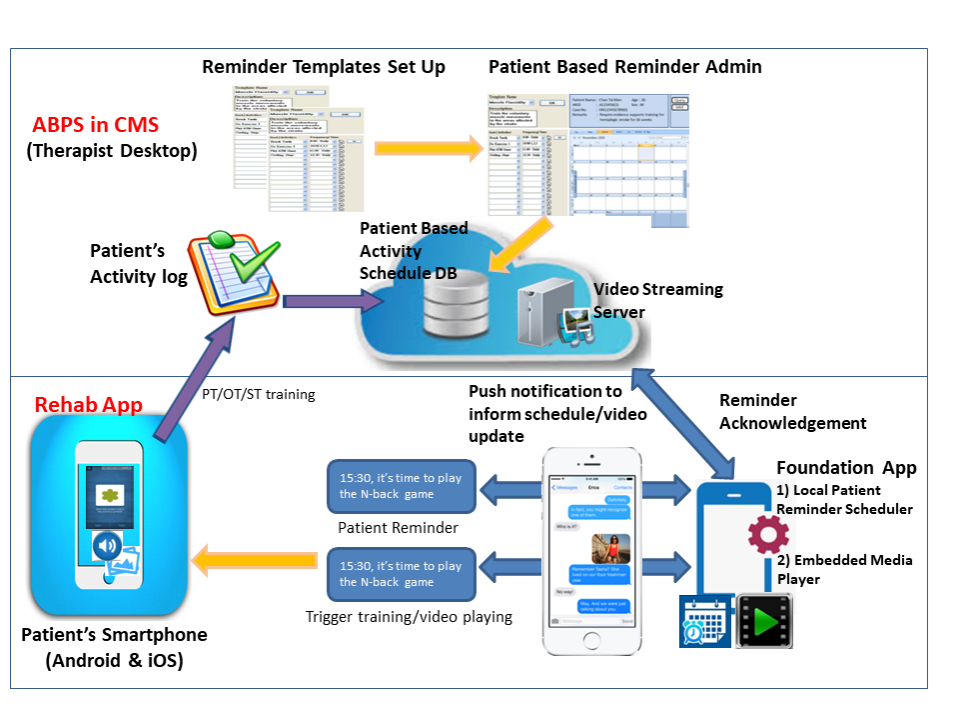
System design. ABPS: Activity Based Prescribing System; Clinical Management System; DB: database; OT: occupational therapist; PT: physiotherapist; ST: speech therapist.

After thorough discussions, it was decided that a new prescription platform and a mobile app would be developed. The utilization of a mobile app in tele-rehabilitation has been supported in many studies [30,31]. After therapist assessment of a patient, exercise videos and reminders could be prescribed through a prescription platform, and the patient could access the prescription through the mobile app. We determined that the prescription platform should be easily accessible and align well with existing clinical workflow of therapist, and the mobile app should be user friendly to older adult users and able to capture the patient’s performance and can channel the results of training back to prescribing therapist for treatment evaluation and planning. The team finally concluded upon a design based on these collective requirements (Figure 1)

### The Activity-Based Prescribing System

The Clinical Management System is the electronic medical record that all therapists in Hospital Authority use daily for clinical practice to disseminate health care information or clinical data and enhance patient care [35]. The activity-based prescribing system (ABPS) was specifically built to integrate into the Clinical Management System; the therapist needed no further log-ins and could view patient information, perform electronic documentation, and prescribe rehabilitation activities on the same platform. The Clinical Management System user password also contains information on the user’s profession; therefore, the system only displayed prescription material specific to that profession. Page tabs built into the ABPS were designed to follow the workflow sequence of therapist (Multimedia Appendix 1): New activity, for videos and reminders selection; History, where all prescribed activities to the patient are displayed and therapist can choose to repeat an order if necessary; Template, which allowed the therapist to prescribe preset personal or departmental templates; Patient-Based Calendar, where the therapist can view all prescribed activities to patients at a glance (this allowed better distribution of patient schedule and prevents overlapping of prescription); Prescribed Activities, for allocating appropriate parameters to the prescription such as treatment period, frequency, and timeslot; and Performance, for therapists to view the performance of a patient for prescribed training.

A therapist could complete a prescription with a few clicks. Altogether, 144 videos were incorporated into the ABPS. The ABPS was designed as a generic prescription platform to allow the future addition of training videos and reminders and future inclusion of more allied health professions.

### The Rehabilitation App

**Figure 2:**
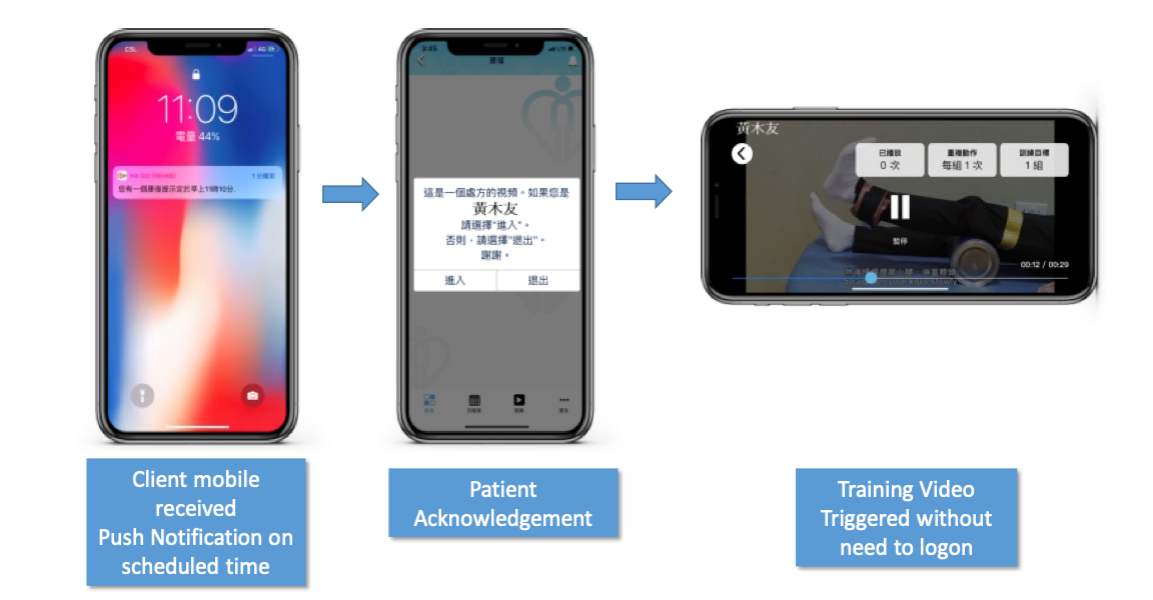
Push notification to trigger training video.

Patients using tele-rehabilitation could be older adults who may have cognitive impairment or poor memory. Thus, the mobile app was designed to be simple and barrier-free. If a therapist prescribed a training video to a patient, a notification would be pushed to the app at the prescribed time. A swipe on the notification message could trigger the training video without having to log in to the app (Figure 2). The patient could also receive reminders—to wear a splint, wear a pressure garment, carrying out oral hygiene, or use walking aids—on a regular basis. Moreover, a daily and weekly activity page was included to facilitate the patient’s viewing of their own rehabilitation schedule. Visual encouragement in the form of a thumbs-up was displayed on the app if the patient completed all prescribed training activities (Multimedia Appendix 1).

### Staff Engagement Strategy and Technical Support

Occupational therapist, physiotherapist, and speech therapist staff committees were engaged to encourage therapists to participate in design, testing, and use. Senior management also expressed that tele-rehabilitation was a corporate direction, and therapists were encouraged to use this new technology. During the rollout of tele-rehabilitation, onsite support was provided to all hospitals. In addition, user guides and support hotline were provided to therapists and training videos were made available to patients to ensure adequate support to both therapists and patients.

### Privacy and Data Security

Privacy and data security were essential concerns in the development of tele-rehabilitation [7]. Data on the tele-rehabilitation platform were encrypted and stored on servers with restricted access. Security scanning was performed according to Hospital Authority standardization and regulation. Servers and databases were hardened for security, and firewall protection was also implemented. Even though the app was designed for easy access, this mode only allowed the patient to view training videos. If a patient needed to access their calendar or other app functions, full log in was still required. This approach balanced quick access to training with maintaining privacy. Moreover, push notifications on mobile phone was generic, without patient condition information or training details.

### Data

We compared tele-rehabilitation use before and during the COVID-19 outbreak. Analysis before the outbreak analysis pertained to the period from October 2019 to January 2020, and analysis during outbreak period analysis pertained to the period from February 2020 to July 2020.

We collected 5 outcomes: throughput, the prescription rate of tele-rehabilitation; patient demographics; patient conditions for which tele-rehabilitation was prescribed; utilization rates by occupational therapists, physiotherapists, and speech therapist; and staff and patient satisfaction.

Satisfaction surveys were prepared and forwarded to both therapists and patients for collecting their opinion on the Rehabilitation App. The format of the surveys was discussed in the focus group. Therapists suggested that the surveys should be simple and require only a short time to complete. The survey for the therapists consisted of 8 questions while the survey for patients consisted of 4 questions. A 5-point scale was use in the survey (1, strongly disagree; 2, disagree; 3, neutral; 4, agree; 5, strongly agree). A prompt was shown on ABPS 30 days after the therapist started prescribing with the platform. The prompt contained a reminder to complete the survey. For patients, 7 days before their prescribed rehabilitation activity ended, a prompt was shown in Rehabilitation App to invite the patient to complete the survey.

### Statistical Analysis

Patient demographic, workforce, and patient condition variables were nonparametric categorical data; therefore, chi-square analysis was used. *P* values less than .05 were statistically significant. When significance was found, the adjusted residual value was calculated. Statistical significance was set as <–1.96 and >1.96 (95% confidence interval). All data were analyzed using SPSS statistical software (version 26; IBM Corp).

## Results

### Impact of the COVID-19 Outbreak

Physiotherapy added 41 musculoskeletal training videos in early March and 15 additional musculoskeletal training videos in April. Speech therapy added 8 swallowing training videos in mid-March. Occupational therapy added 8 pulmonary training videos in early April. A total of 72 videos were added from February to April.

### Throughput Analysis

**Figure 3:**
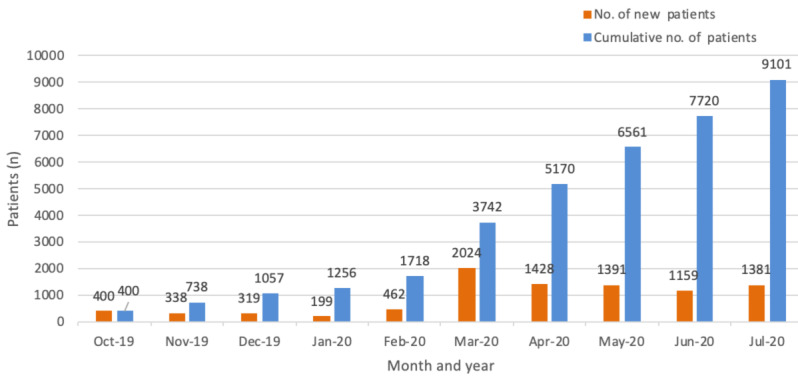
Patients prescribed tele-rehabilitation by month.

**Figure 4:**
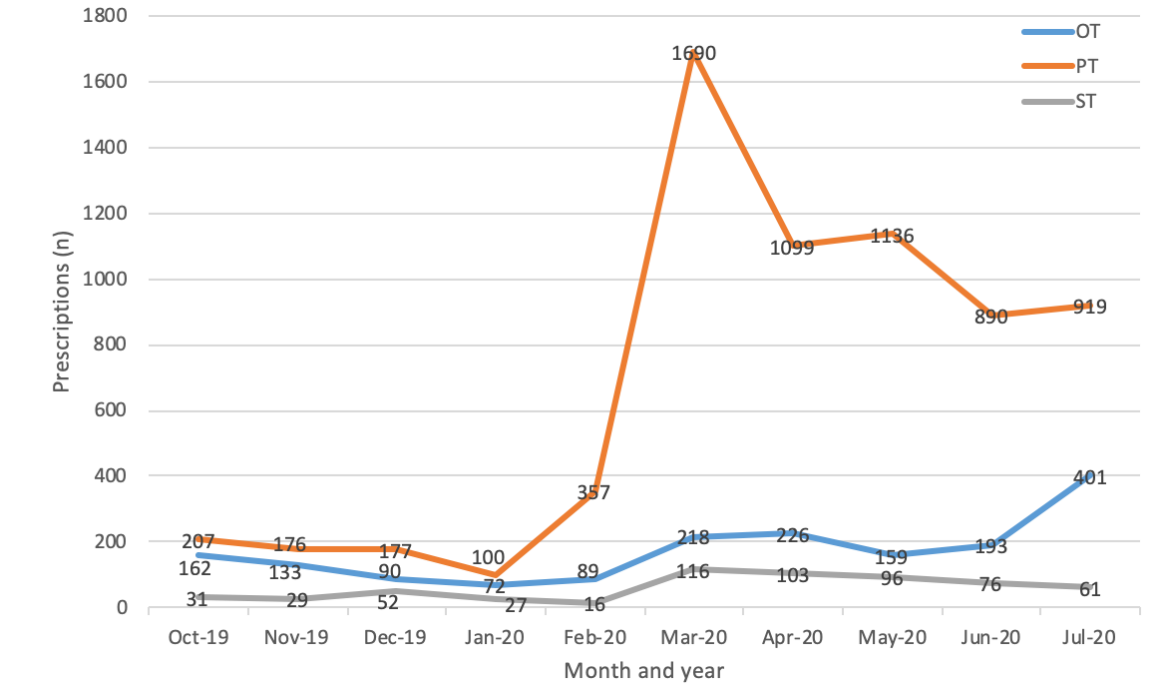
Tele-rehabilitation prescriptions per month by professions.

The number of prescriptions per month showed a slightly decrease from October 2019 to January 2020. The number of new patients per month increased to 462 in February 2020 and spiked to 2024 in March 2020. The total number of patients prescribed accumulated to 9101 (Figure 3) by the end of July 2020. The prescription trend was stable in the months from May to July. Physiotherapists exhibited the highest increase in tele-rehabilitation prescriptions (Figure 4). Up until the end of July 2020, a total of 131995 training videos were prescribed, and the overall adherence rate of patients was 81.7% (107,840/131,995).

### Patient Demographics

**Figure 5:**
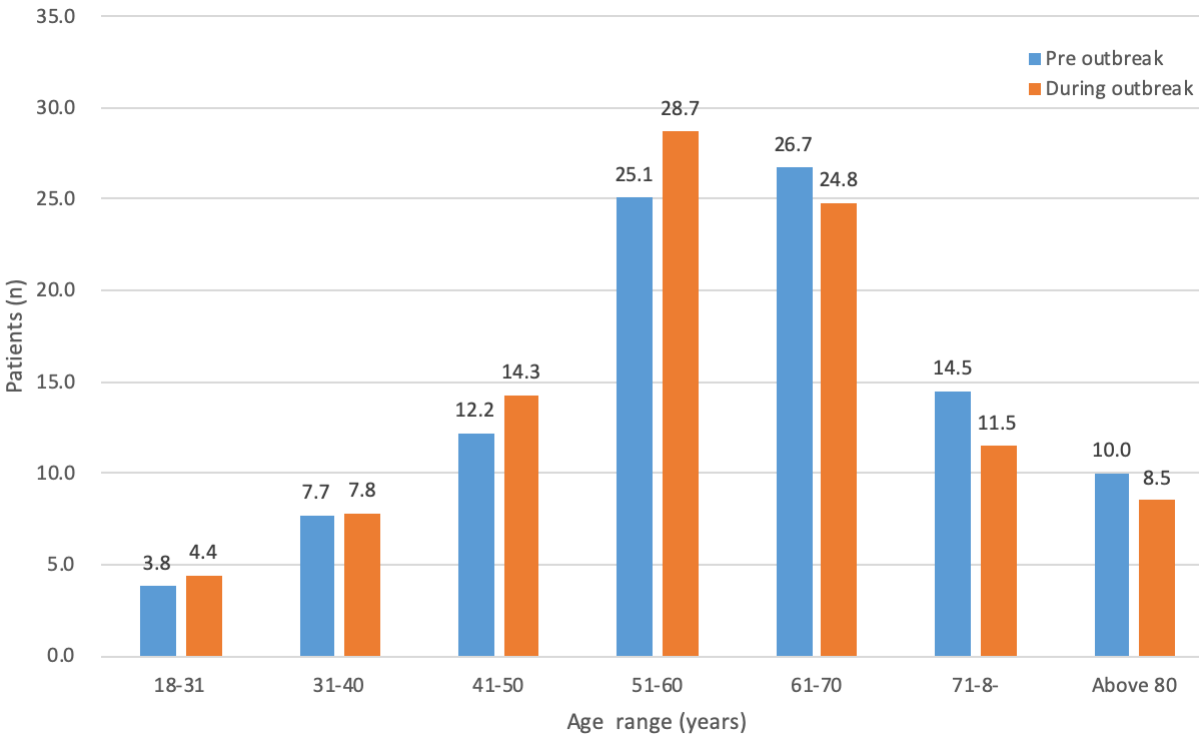
Before and during outbreak age distributions of patients prescribed tele-rehabilitation.

The Rehabilitation App was designed for adult patients, and the age of prescribed patients ranged from 18 to 106 years old. Age group analysis of patients revealed that before the outbreak, the age group with the highest prescription rate was 61 to 70 years (Figure 5). During the outbreak the age group with the highest prescription rate was 51 to 60 years. Before the outbreak, 48.8% of patients (610/1246) were below 60 years of age. After the outbreak, 55.2% of patients (4001/7845) were below 60 years of age (Table 1). There was no statistically significant difference in gender (*P*=.83) or age distribution (above and below 60 years: *P*=.36).

**Table 1:** Tele-rehabilitation patient demographics before and during the COVID-19 outbreak.

Characteristic			Before^a^ (n=1246)		During^b^ (n=7845)
**Gender, n (%)**					
	Female	648 (52.0)		4158 (53.0)	
	Male	598 (48.0)		3687 (47.0)	
**Age (years), n (%)**					
	Below 60	610 (49)		4001 (55)	
	Above 60	636 (51)		3884 (45)	
Age (years), mean (SD)			60 (15.5)		59 (16.2)
Age (years), median			61		60

^a^October 2019 to January 2020.

^b^February 2020 to July 2020.

### Patient Conditions

#### Speech Therapy

**Figure 6:**
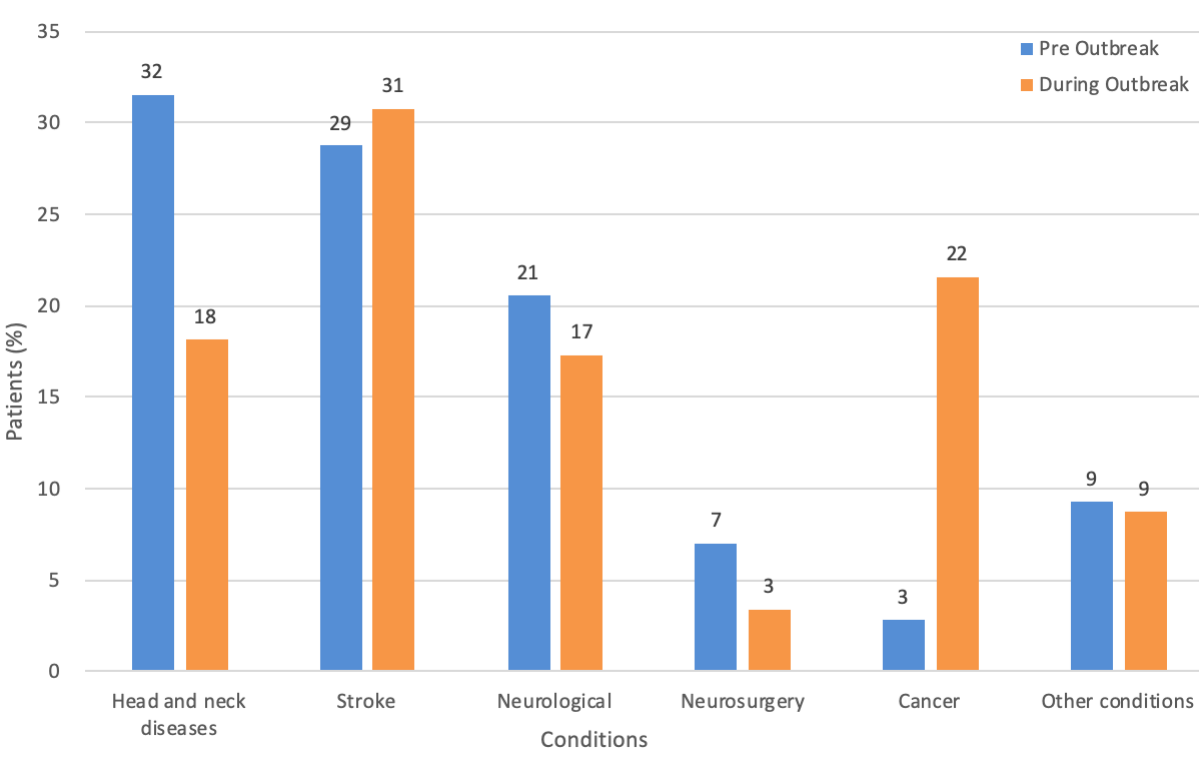
Percentage distribution of patient conditions for speech therapy before and during the outbreak.

Speech therapy had a relatively simple patient condition distribution; the main conditions were head and neck diseases, stroke, neurological conditions, neurosurgery, and cancer. Stroke and head and neck disease remained the largest case group for speech therapy throughout (Figure 6). The mean number of patients per month prescribed tele-rehabilitation before the outbreak was 35.5; whereas the mean increased to 117 during the outbreak (Table 2). There was increase in prescription per month (230%; ie, 117 – 35.5 / 35.5). Patient condition distributions before and during the outbreak did not significantly differ (*P*=.998).

**Table 2:** Patient conditions for which speech therapy was prescribed before and during the COVID-19 outbreak.

Patient conditions	Before^a^			During^b^	
	Mean per month (SD)	%	Mean per month (SD)		%
Head and neck conditions	11.2 (3.4)	31.5	21.3 (5.9)		18.2
Stroke	10.2 (1.3)	28.7	36.0 (6.6)		30.7
Neurological	7.3 (1.2)	20.6	20.3 (6.1)		17.4
Neurosurgery	2.5 (0.8)	7.0	4.0 (2.4)		3.3
Cancer	1.0 (1.8)	2.8	25.3 (7.1)		21.6
Other conditions	3.3 (0.7)	9.3	10.3 (3.8)		8.8
Total	35.5	100	117.0		100

^a^October 2019 to January 2020.

^b^February 2020 to July 2020.

#### Occupational Therapy

**Figure 7:**
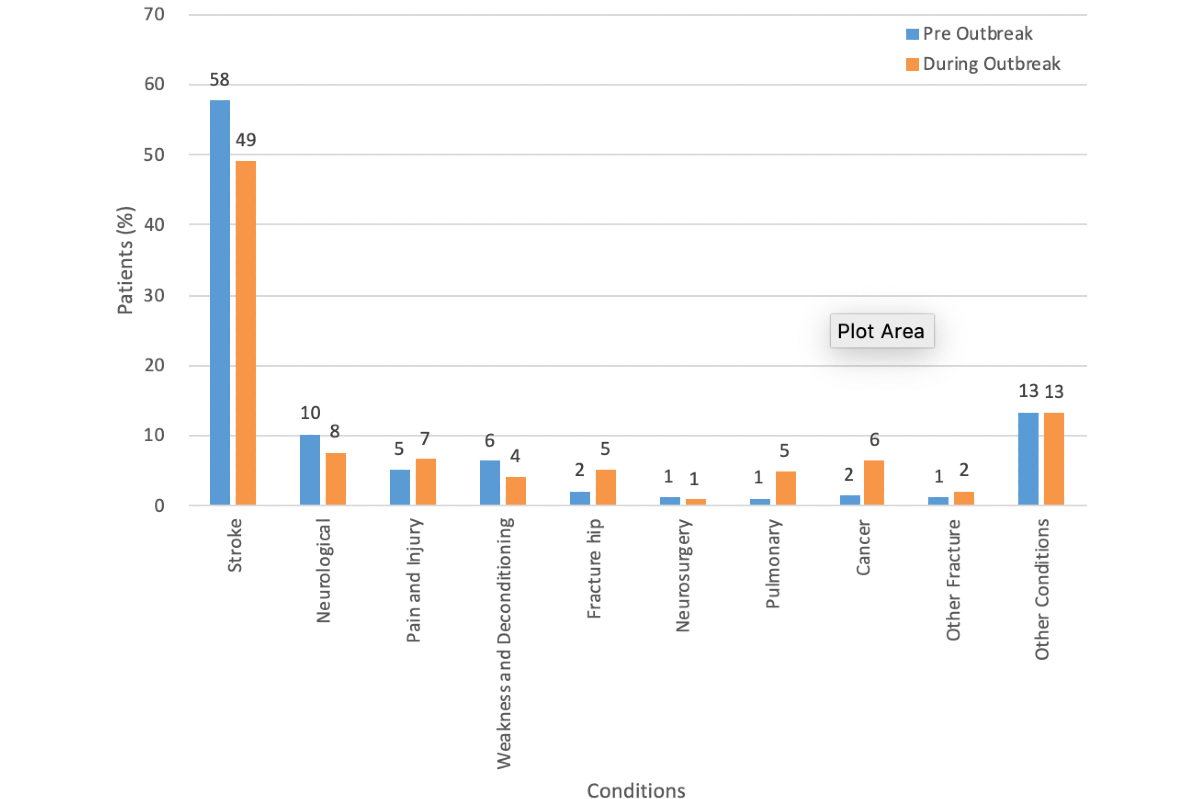
Percentage distribution of patient conditions before and during outbreak for occupational therapy.

Occupational therapy had several major patient condition groups including stroke, neurological conditions, weakness and deconditioning, pain and injury, cancer, and fractures (Figure 7). Stroke-related conditions constituted over 50% of total prescriptions (68/117.9), whereas fracture hip constituted only 2% (2.5/117.9). The mean per month before the outbreak was 118 patients, whereas the mean per month during the outbreak was 214 patients. There was increase in prescription per month (81.8%; ie, 214.3 – 117.9 / 117.9) (Table 3). Patient condition distributions before and during the outbreak did not significantly differ (*P*=.93).

**Table 3:** Patient conditions for which occupational therapy was prescribed before and during the COVID-19 outbreak.

Patient conditions	Before^a^			During^b^	
	Mean per month (SD)	%	Mean per month (SD)		%
Stroke	68.0 (13.0)	57.7	105.3 (32.1)		49.1
Neurological	12.0 (1.9)	10.2	16.3 (6.6)		7.6
Pain and injury	6.0 (0.5)	5.1	14.2 (4.7)		6.6
Weakness and deconditioning	7.5 (0.8)	6.4	8.7 (3.9)		4.1
Fracture hip	2.5 (0.5)	2.1	11.0 (3.9)		5.1
Neurosurgery	1.5 (1.9)	1.3	1.8 (1.8)		0.8
Pulmonary	1.3 (0.5)	1.1	10.3 (6.8)		4.8
Cancer	1.8 (1.9)	1.5	13.8 (4.9)		6.4
Other fracture	1.5 (0.6)	1.3	4.5 (2.3)		2.1
Other conditions	15.8 (2.3)	13.4	28.3 (8.1)		13.2
Total	117.9	100	214.3		100

^a^October 2019 to January 2020.

^b^February 2020 to July 2020.

#### Physiotherapy

**Figure 8:**
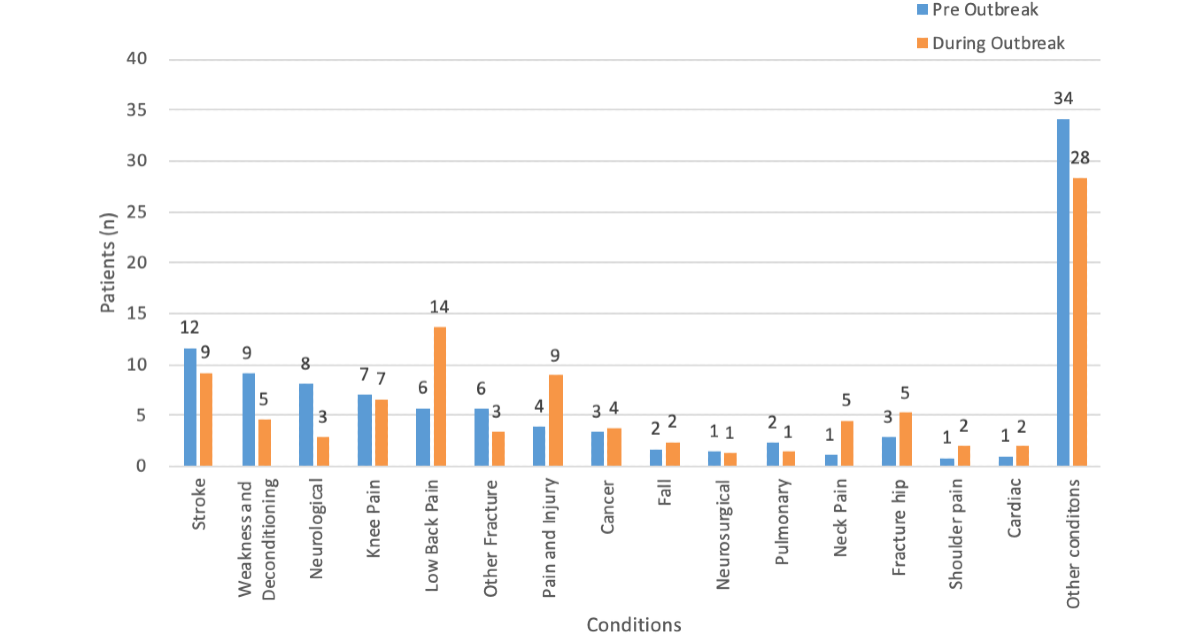
Percentage distribution of patient conditions before and during outbreak for physiotherapy.

Physiotherapy had a diverse patient conditions distribution. There were several conditions including stroke, weakness and deconditioning, fracture hip, pain and injury, neurological conditions, and low back pain (Figure 8). The most prescribed condition before outbreak was stroke (20.3/173.9, 12%), whereas the most prescribed condition during the outbreak was lower back pain (138.5/1015.2, 14%). Other than clinical conditions related to frail older adult patients, hip fracture only constituted 3% (5/173.2) before the outbreak and 5% (53.2/1015.2) during the outbreak. There were many conditions related to musculoskeletal problems; *other conditions* comprised a large variety of musculoskeletal conditions including soft tissue problems and degenerative problems and occupied the highest percentage before and during the outbreak. The mean number of prescriptions per month before the outbreak was 174, and the mean increased to 1015 per month during the outbreak. The average number of prescription per month increased after the outbreak (484%; ie, 1015 – 174 / 174) (Table 4). There was a statistically significant difference between patient condition distributions before and during the outbreak (*P*=.04). There were statistically significant decreases in weakness and deconditioning (adjusted residual –2.5, 2.5) and neurological condition (adjusted residual –3.4, 3.4); (2) statistically significant increase in lower back pain (adjusted residual –2.9, 2.9), pain and injury (adjusted residual –2.2, 2.2), and neck pain (adjusted residual –2.1, 2.1). This was also demonstrated by the percentage change in distribution in these conditions.

**Table 4:** Patient conditions for which physiotherapy was prescribed before and during the COVID-19 outbreak.

Patient conditions	Before^a^			During^b^			Adjusted residual value^c^
	Mean per month (SD)	%	Mean per month (SD)		%		
Stroke	20.3 (6.0)	11.7	93.3 (13.4)		9.2	–0.9, 0.9	
Weakness and deconditioning	15.8 (2.7)	9.1	46.8 (4.6)		4.6	–2.5, 2.5^c^	
Neurological	14.0 (2.9)	8.0	29.2 (9.9)		2.9	–3.4, 3.4^c^	
Knee pain	12.3 (2.7)	7.1	67.0 (39.3)		6.6	–0.1, 0.1	
Lower back pain	10.0 (5.5)	5.7	138.5 (62.8)		13.7	–2.9, 2.9^c^	
Other fracture	9.8 (4.2)	5.6	34.2 (16.6)		3.4	–1.5, 1.5	
Pain and injury	6.8 (3.9)	4.0	90.3 (45.1)		8.9	–2.2, 2.2^c^	
Cancer	5.8 (2.7)	3.3	37.7 (10.4)		3.7	–0.2, 0.2	
Fall	3.0 (1.2)	1.7	24.2 (6.5)		2.4	–0.5, 0.5	
Neurosurgical	2.5 (2.1)	1.4	13.3 (8.3)		1.3	–0.5, 0.5	
Pulmonary	4.0 (0.5)	2.3	14.3 (5.9)		1.4	–0.9, 0.9	
Neck pain	2.0 (1.1)	1.1	46.0 (27.0)		4.5	–2.1, 2.1^c^	
Fracture hip	5.0 (1.9)	2.9	53.2 (13.5)		5.2	–1.3, 1.3	
Shoulder pain	1.5 (0.7)	0.9	20.3 (5.5)		2.0	–0.8, 0.8	
Cardiac	1.8 (1.1)	1.0	19.5 (6.0)		1.9	–0.8, 0.8	
Other conditions	59.3 (20.4)	34.1	287.3 (63.3)		28.3	–1.5, 1.5	
Total count	173.9	100	1015.2		100	N/A^d^	

^a^October 2019 to January 2020.

^b^February 2020 to July 2020.

^c^Statistically significant 95% confidence interval (<–1.96 and >1.96).

^d^N/A: not applicable.

### Workforce

In February 2020, there were a total of 907 occupational therapists, 1177 physiotherapists, and 112 speech therapists employed in Hospital Authority, and 1112 therapists (372 occupational therapists, 635 physiotherapists, and 105 speech therapists) prescribed tele-rehabilitation to patients, which constituted 50.6% (1112/2196) of the total workforce. Physiotherapy and occupational therapy had a 3-tier rank structure (rank I, rank II, and senior). Rank II was the entry rank, and rank I was the middle rank. Speech therapy had a 2-tier rank structure (basic and senior). Speech therapists had the highest overall prescription rate (speech therapists: 105/112, 93.8%; physiotherapists: 635/1177, 54.0%; occupational therapists: 372/907, 41.0%). There were statistically significant differences in prescriptions by rank for occupational therapists (*P*=.001) and physiotherapists (*P*<.001), and no statistically significant difference for speech therapists (*P*=.45). Further analysis by adjusted residual value demonstrated that there were differences in prescriptions between occupational therapist II and senior occupational therapist and between physiotherapist II, physiotherapist I, and senior physiotherapist (Table 5).

**Table 5:** Analysis of prescription according to therapist rank.

Rank			Total workforce		Prescribed tele-rehabilitation		Percentage of workforce		Adjusted residual value		*P* value
**Occupational therapy**											.001
	Occupational therapist II	442		201		45.5		–2.7, 2.7			
	Occupational therapist I	389		154		39.6		–0.8, 0.8			
	Senior occupational therapist	76		17		22.4		–3.5, 3.5			
**Physiotherapy**											<.001
	Physiotherapist II	554		350		63.2		–6.0, 6.0			
	Physiotherapist I	524		266		50.8		–2.0, 2.0			
	Senior physiotherapist	99		19		19.2		–7.3, 7.3			
**Speech therapy**											.45
	Speech therapist	104		97		93.3		–0.8, 0.8			
	Senior speech therapist	8		8		100		–0.8, 0.8			

### Therapist and Patient Satisfaction

Of 2196 therapists, 111 therapists completed the survey; the response rate was 5.2%. Overall satisfaction toward the Rehabilitation App was rated as 3.7 (Table 6). It was opined, by therapists, that they needed to use a considerable amount of time to instruct and assisted patients to install the Rehabilitation App. The preparation work was regarded as increased workload to therapists. In addition, several meetings with therapists revealed that they required an expanded video library in order to prescribe training to patients with a variety of conditions. The survey showed that therapists found the Rehabilitation App to be effective for patients to continue rehabilitation in a home setting.

**Table 6:** Therapist survey scores.

Therapist questions	Score (n=111), mean
The installation procedures are easy to administer	3.5
The training app is well organized.	3.8
The training app is user-friendly	3.6
The content of the app meets the patient’s training need	3.7
The app can enhance patient’s treatment frequency apart from regular treatment	3.8
The app could facilitate you to prescribe the home program	4.2
The app could assist you in treatment planning	3.8
Overall, you satisfy with the training app.	3.7

The response from the patient’s side was very positive. The response rate was 28.8%, with 2623 of 9101 patients completing the survey. Overall satisfaction rate was rated as 4.2 (Table 7). Several commendation letters were received regarding the Rehabilitation App, and most patients found the app to be user friendly and helpful.

**Table 7:** Patient survey scores.

Patient questions	Score (n=2623), mean
The training app is easy to use.	4.2
The training app improves my participation in the home program.	4.2
The training app is helpful for my rehabilitation.	4.1
Overall, I am satisfied with the training app.	4.2

## Discussion

### Principal Results

Use of tele-rehabilitation increased remarkably during the COVID-19 pandemic. Physiotherapy had the highest number of prescriptions. Tele-rehabilitation was mostly prescribed to patients between 51 and 70 years of age. Patients reported a high level of satisfaction. Over 50% of the total workforce prescribed tele-rehabilitation to patients (1112/2196, 50.6%). Originally, tele-rehabilitation was designed to treat patients with stroke, patients with hip fracture, and older adults with frailty. Our study showed that tele-rehabilitation can be used for a much wider spectrum of patient conditions. The generic design of the tele-rehabilitation was able to expand training content and cope with the service demand for rehabilitation during the outbreak period.

### Utilization of Tele-rehabilitation Before and During Outbreak

Tele-rehabilitation utilization reached a peak in March 2020 during the first wave of outbreak in Hong Kong. Tele-rehabilitation use dropped from 2024 new patients in March to approximately 1300 per month from April to July. The stable trend indicated that tele-rehabilitation was used irrespective of number of confirmed COVID-19 cases. Continuous monitoring is needed to study the sustainability of utilization and especially during the postpandemic phase.

Our study showed that there was no difference in distribution between patients above or below 60 years old. This finding echos those of Crotty et al [34]—the age of patient was not really a barrier for the acceptance of tele-rehabilitation. For the 2623 patients who responded to the survey, overall satisfaction score for the app was a mean of 4.2 out of 5. Moreover, the overall adherence rate for tele-rehabilitation in our study was recorded at a satisfactory level of 81.7 % (107,840/131,995). This provided a reliable reflection of the high acceptance of tele-rehabilitation by patients. On the other hand, only 111 therapists responded to the survey, and the overall satisfaction score of therapists for the app was a mean of 3.73 out of 5. The design of the survey questions for therapists had a serious shortcoming in that it focused on the app rather than on the ABPS. It was inappropriate for therapists to provide opinions on using the app. A more comprehensive and appropriately designed survey would be needed to reflect the opinion of therapists on ABPS.

Analysis of workforce data demonstrated that 50.6% of the total workforce (1112/2196) prescribed tele-rehabilitation. There was significant difference in prescription rate between basic and senior ranks in occupational therapy (*P*=.001) and physiotherapy (*P*<.001), and there was an apparent difference in the prescription rate of tele-rehabilitation among the 3 allied health professions (occupational therapist: 372/907, 41.0%, physiotherapist: 635/1177, 54%, speech therapist: 105/112, 93.8%). Speech therapy had the most severe disruption in service during the outbreak, which could be attributed to the high rate of prescription. There was a highest absolute number of prescriptions among physiotherapist. This was attributable to fact that physiotherapy had the largest workforce, and there was extensive use of tele-rehabilitation for musculoskeletal conditions. The differences, however, also raised the question of whether tele-rehabilitation was equally suitable to different allied health services. For example, tele-rehabilitation in the form of a video may not fit activities of daily living training, which requires the use of tools and equipment. Whereas for physical training prescribed by physiotherapist and oral-motor training prescribed by speech therapists, video training is a suitable format.

Analysis of clinical conditions revealed that there was an increase in prescriptions for patients after stroke during the outbreak period (1.55-fold increase in occupational therapy, 4.60-fold increase in physiotherapy, and 3.53-fold increase in speech therapy). These findings aligned well with the initial goals of the tele-rehabilitation platform. However, we noticed that hip fracture ranked rather low in the prescription rate for both physiotherapists and occupational therapists which was surprisingly not aligned with the objectives of the platform’s development. On the other hand, both physiotherapists and occupational therapists prescribed tele-rehabilitation for a broad spectrum of clinical conditions. There was a significant increase in prescriptions to musculoskeletal conditions of lower back pain (adjusted residual –2.9, 2.9), neck pain (adjusted residual –2.1, 2.1), and pain and injury (adjusted residual –2.2, 2.2). The results demonstrate that tele-rehabilitation is indicated for a broad spectrum of patient conditions.

### Tele-rehabilitation System Design

A generic design was adopted for both the ABPS and mobile app. This facilitated rapid expansion of training content. Previous studies [10,19,21,26,31] on tele-rehabilitation often require the use of sophisticated communication tools, equipment, or software. During the COVID-19 crisis, the use of off-the-shelf technology and the expansibility of our tele-rehabilitation design enabled provision of rehabilitation service to a large amount of patients. In addition, Hong Kong has one of the highest smartphone ownership rates in Asia—for Hong Kong citizens over 10 years old, 88% of females and 91% of males own a smartphone [36]. This high ownership of smartphones could be a facilitating factor for our tele-rehabilitation platform use.

### Opportunities and Challenges

The COVID-19 pandemic has altered health care delivery globally. Severe restrictions such as social distancing and the suspension of rehabilitation services were enacted to prevent spread of disease. The World Health Organization recommended postponing treatments that were not considered urgent in order to ensure safety, while still guaranteeing the essential rehabilitation services [37]. The pandemic has catalyzed the rapid adoption of telehealth worldwide [38]. Tele-rehabilitation is promising for overcoming service disruption during the outbreak [39,40]. Implementation of tele-rehabilitation has been recommended by different allied health professions [39-42].

Through the advent of technology, faster internet connection, cheaper smart devices (smartphones and tablets), and new software being available, tele-rehabilitation is able to offer many benefits. However, there are challenges ahead if tele-rehabilitation is to be used extensively in the future. For example, the use of tele-rehabilitation is a paradigm shift for therapists from conventional face-to-face interventions. During the outbreak, there was a rapid increase in the number of therapists who needed to prescribe tele-rehabilitation. Consequently, training and accrediting staff to use tele-rehabilitation became essential. A *train the trainer* model can be a feasible to allow rapid staff development to enable trained staff to onboard others in the use of tele-rehabilitation [39]. There was concern from Hospital Authority that the number of infections from the pandemic may fluctuate and the pandemic could last for some time and that there would be high utilization of tele-rehabilitation. Consequently, training and support for therapists using tele-rehabilitation was considered important. As many allied health professions are predominantly hands-on and skill-based professions, the lack of physical contact with patients is a hurdle for tele-rehabilitation utilization. Thus, essential infrastructure enhancements for future tele-rehabilitation development include patient evaluation, assessment, physiological monitoring, and education [39]. Moreover, legislation and payment arrangement should be in place to facilitate tele-rehabilitation delivery [38,39].

The COVID-19 pandemic will not affect the acute outbreak period alone but may also create a serious backlog for rehabilitation in the postpandemic recovery period, which is referred to as “care debt [38].” To transform tele-rehabilitation from crisis mode during pandemic to a sustainable mode after outbreak requires clear deliberation and planning.

### Limitations

This observational study has a number of limitations. This study only reports outcomes of tele-rehabilitation utilization before and during outbreak periods. It does not cover the clinical effectiveness of tele-rehabilitation to patients; this requires additional well-powered clinical studies. The Unified Theory of Acceptance and Use of Technology [12] is used as a guiding framework for development of the tele-rehabilitation. It is a limitation that this model is not used to evaluate the acceptance of this new technology. The design of the survey questions also had serious shortcoming. The study period was relatively short, and sustained utilization of tele-rehabilitation requires a longer duration study. More meaningful information can be gathered if the study is extended to the period when COVID-19 pandemic is over.

### Conclusions

The COVID-19 pandemic has seriously affected rehabilitation service delivery. Our study has shown that a tele-rehabilitation platform was used extensively and effectively during the outbreak period to mitigate service disruption. In addition to the original targeted conditions (stroke and hip fracture), tele-rehabilitation was prescribed for a large variety of clinical conditions. The tele-rehabilitation platform, though it cannot replace all face-to-face rehabilitation services, has demonstrated its potential during the COVID-19 crisis and has promising potential to become a sustainable service delivery model.

## Appendix

Multimedia Appendix 1 Supplementary material.

## Abbreviations

ABPS: activity-based prescribing system
