# Supplementary material for: Tele-Rehabilitation to Combat Rehabilitation Service Disruption During COVID-19 in Hong Kong: Observational Study
Source: JMIR Rehabil Assist Technol. 2021 Aug 19;8(3):e19946. doi: 10.2196/19946 (PMC8396543; doi:10.2196/19946)

**Appendix**

Appendix 1. New Activity page of ABPS (Therapist can add video by pressing + icon)


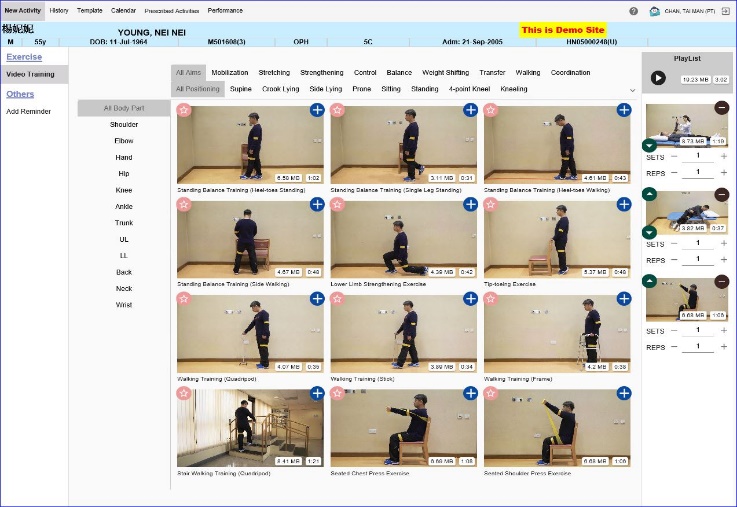


Appendix 2. History page of ABPS


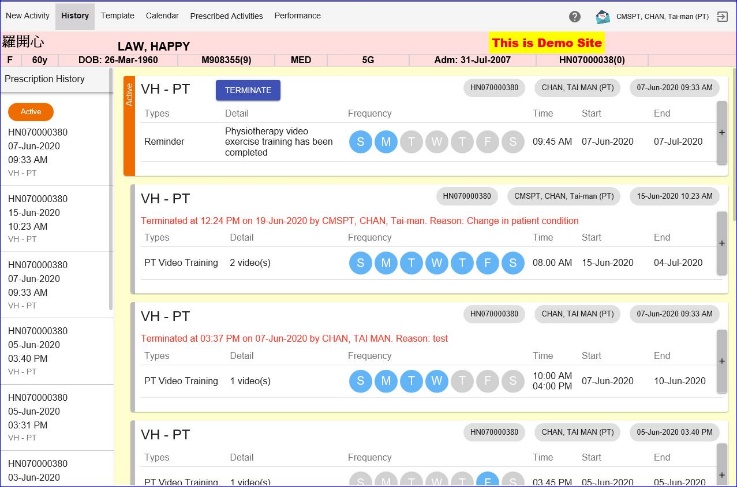


Appendix 3. Template page of ABPS


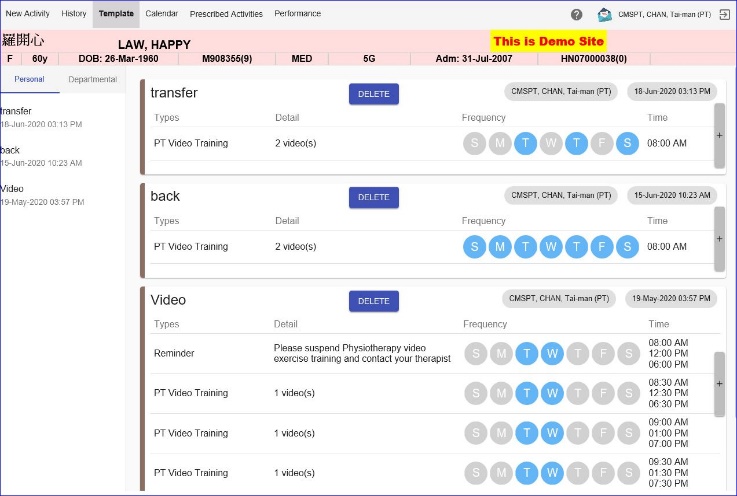


Appendix 4. Calendar page of ABPS


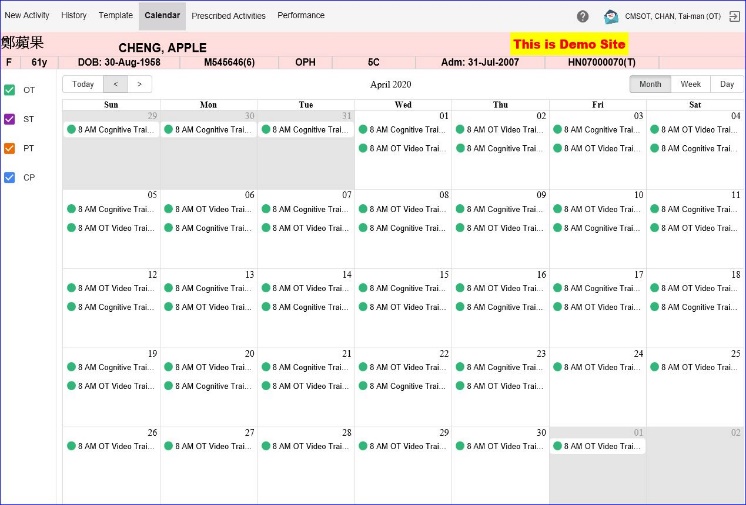


Appendix 5. Prescribed Activities page of ABPS


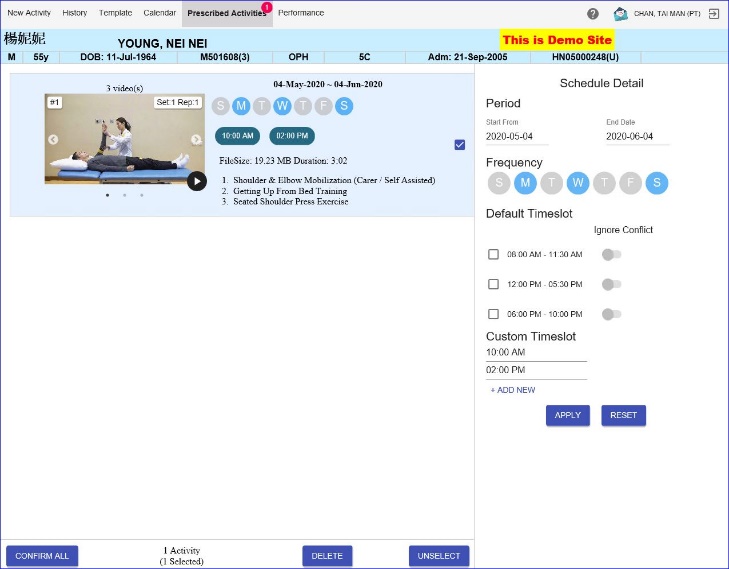


Appendix 6. Performance page of ABPS


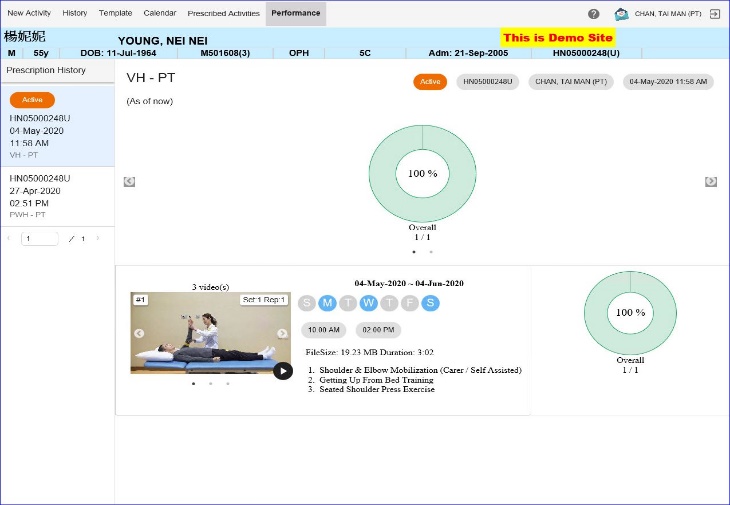


Appendix 7: Thumb up encourage to patient


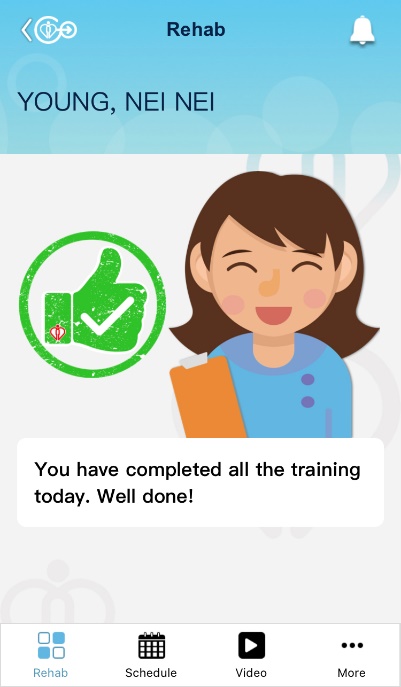

Supplement: Multimedia Appendix 1 [file rehab_v8i3e19946_app1.docx]
